# The implementation of an inflammatory bowel disease-specific enhanced recovery after surgery protocol: an observational cohort study

**DOI:** 10.1007/s10151-024-02933-3

**Published:** 2024-05-25

**Authors:** V. Lin, J. K. Poulsen, A. F. Juvik, O. Roikjær, I. Gögenur, T. Fransgaard

**Affiliations:** 1 Center for Surgical Science, Zealand University Hospital Koge, Lykkebækvej 1, 4600 Køge, Denmark; 2 Department of Surgery, Zealand University Hospital Koge, Køge, Denmark; 3 Department of Clinical Medicine, University of Copenhagen, Copenhagen, Denmark

**Keywords:** Surgery, IBD, ERAS, Complications

## Abstract

**Background:**

The implementation of Enhanced Recovery After Surgery (ERAS) protocols has resulted in improved postoperative outcomes in colorectal cancer surgery. The evidence regarding feasibility and impact on outcomes in surgery for inflammatory bowel disease (IBD) is limited.

**Methods:**

We performed a retrospective observational cohort study, comparing patient trajectories before and after implementing an IBD-specific ERAS protocol at Zealand University Hospital. We assessed the occurrence of serious postoperative complications of Clavien-Dindo grade 3 or higher as our primary outcome, with postoperative length of stay in days and rate of readmissions as secondary outcomes, using χ^2^, Mann–Whitney test, and odds ratios adjusted for sex and age.

**Results:**

From 2017 to 2023, 394 patients were operated on for IBD and included in our study. In the ERAS cohort, 39/250 patients experienced a postoperative complication of Clavien-Dindo grade 3 or higher compared to 27/144 patients in the non-ERAS cohort (15.6% vs. 18.8%, *p* = 0.420) with an adjusted odds ratio of 0.73 (95% CI 0.42–1.28). There was a significantly shorter postoperative length of stay (median 4 vs. 6 days, *p* < 0.001) in the ERAS cohort compared to the non-ERAS cohort. Readmission rates remained similar (22.4% vs. 16.0%, *p* = 0.125).

**Conclusions:**

ERAS in IBD surgery was associated with faster patient recovery, but without an impact on the occurrence of serious postoperative complications and rate of readmissions.

## Introduction

The implementation of Enhanced Recovery After Surgery (ERAS) principles in colorectal cancer surgery has led to faster patient recovery, improved surgical outcomes, and reduced healthcare costs [1–4]. Rather than using specific interventions, it consists of a multimodal approach before, during, and after surgery aimed at reducing the surgical stress response and maintaining physiological functions [5].

The evidence regarding adoption of ERAS for patients with inflammatory bowel disease (IBD) is limited, and protocols are often based on those for patients with colorectal cancer, despite some important differences in these patient groups [6–9]. The preoperative use of long-term immunomodulating medications and malnutrition due to chronic inflammation warrant IBD-specific ERAS protocols, as patients undergoing surgery for Crohn’s disease and ulcerative colitis have a high risk of postoperative complications [10, 11].

We assessed the impact of implementing IBD-specific ERAS principles in patients undergoing surgery for Crohn’s disease and ulcerative colitis at our institution. Our primary outcome was the occurrence of postoperative complications of Clavien-Dindo grade 3 or higher [12] and secondary outcomes were postoperative length of stay and readmission rate. We hypothesized that applying ERAS principles to IBD surgery would improve these short-term postoperative outcomes.

## Materials and methods

We performed an observational cohort study, where we compared patients that underwent elective and subacute surgery for IBD before and after the introduction of an IBD-specific ERAS protocol at Zealand University Hospital, a referral center for IBD surgery. Data was collected from December 1, 2017 to September 30, 2023 and entered into an observational database using patients’ electronic health records. There were no missing data.

### Patient population

We included patients over 18 years of age that underwent surgery for Crohn’s disease, ulcerative colitis, and indeterminate IBD at Zealand University Hospital, with a follow-up of 30 days after discharge. Patients that underwent emergency surgery, defined as surgery performed within 24 h of the time of indication for surgery set (e.g., for ileus, bowel perforation, life-threatening bleeding) were excluded. Subacute surgery, e.g.,, for treatment-resistant disease, performed within a few days but more than 24 h after setting the surgical indication, was included because these patients might still benefit from ERAS and could still follow the same intra- and postoperative ERAS principles.

We excluded appendectomies as well as minor local procedures such as perianal fistula surgery.

We recorded patient-specific, IBD-specific, surgery-specific, and postoperative variables (Tables 1, 2, 3).

**Table 1:** Preoperative characteristics of patients included in the IBD surgery database

Preoperative characteristics	Non-ERAS (*n* = 144)	ERAS (*n* = 250)	*p* value
Male sex, *n* (%)	71 (49.3)	136 (54.4)	0.330
Age, median (IQR)	44.0 (30.0–58.3)	49.0 (33.3–61.0)	0.088
BMI, median (IQR)	24.5 (22.7–27.9)	25.0 (22.4–29.3)	0.359
Current smoker, *n* (%)	40 (27.8)	50 (20.0)	0.077
ASA, *n* (%)			
1	5 (3.5)	1 (0.4)	0.016
2	125 (86.8)	199 (79.6)	0.072
3	13 (9.0)	47 (18.8)	0.009
4	1 (0.7)	3 (1.2)	0.630
WHO performance status, *n* (%)			
0	125 (86.8)	222 (88.8)	0.556
1	16 (11.1)	23 (9.2)	0.541
≥ 2	3 (2.1)	5 (2.0)	0.955
Type of IBD, *n* (%)			
Crohn’s disease	102 (70.8)	150 (60.0)	0.031
Ulcerative colitis	40 (27.8)	98 (39.2)	0.022
Indeterminate IBD	2 (1.4)	2 (0.8)	0.574
Disease duration in years, median (IQR)	6.0 (1.0–16.0)	7.0 (2.0–15.0)	0.197
Prior surgery for IBD, *n* (%)	61 (42.4)	85 (34.0)	0.098
Current medical treatment for IBD, *n* (%)			
None	71 (49.3)	89 (35.6)	0.008
Systemic steroids	33 (22.9)	80 (32.0)	0.055
Biologicals	40 (27.8)	85 (34.0)	0.201
Immunosuppressants*	13 (9.0)	17 (6.8)	0.422
5-ASA derivatives	9 (6.3)	20 (8.0)	0.522

*IQR* interquartile range, *BMI*  body mass index, *ASA* American Society of Anesthesiology, *WHO* World Health Organization, *IBD* inflammatory bowel disease, *5-ASA* 5-aminosalicylic acid

*Immunosuppressants include azathioprine and methotrexate

**Table 2:** Intraoperative characteristics of patients included in the IBD surgery database

Intraoperative characteristics	Non-ERAS (*n* = 144)	ERAS (*n* = 250)	*p* value
Surgical priority, *n* (%)			
Elective	99 (68.8)	172 (68.8)	0.992
Subacute	45 (31.2)	78 (31.2)	
Surgery type, *n* (%)			
Open	23 (16.0)	16 (6.4)	0.002
Laparoscopic	120 (83.3)	226 (90.4)	0.039
Of which converted to open	21 (17.5)	11 (4.4)	< 0.001
Local	1 (0.7)	8 (3.2)	0.109
Primary procedure, *n* (%)			
Small bowel resection	7 (4.9)	2 (0.8)	0.009
Ileocolic or right-sided resection	39 (27.1)	59 (23.6)	0.441
Left-sided resection	1 (0.7)	5 (2.0)	0.308
Subtotal colectomy	44 (30.6)	109 (43.6)	0.011
Proctocolectomy	1 (0.7)	3 (1.2)	0.630
Protectomy	5 (3.5)	18 (7.2)	0.129
Stoma takedown	18 (12.5)	18 (7.2)	0.079
Stoma creation without resection	5 (3.5)	2 (0.8)	0.053
Stoma revision	9 (6.3)	13 (5.2)	0.662
Resection of neoterminal ileum	11 (7.6)	17 (6.8)	0.755
Other	4 (2.8)	4 (1.6)	0.425
Creation of ostomy, *n* (%)	80 (55.6)	154 (61.6)	0.239
Creation of anastomosis, *n* (%)	60 (41.7)	82 (32.8)	0.078
Type of anastomosis, *n* (%)			
Ileocolic	51 (85.0)	74 (90.2)	0.341
Ileoileal	6 (10.0)	5 (6.1)	0.390
Ileorectal	1 (1.7)	0	–
Colocolic	1 (1.7)	0	–
Colorectal	1 (1.7)	3 (3.7)	0.479
Anastomotic technique, *n* (%)			
Stapled, linear stapler	50 (83.3)	17 (20.7)	< 0.001
Stapled, circular stapler	2 (3.3)	3 (3.7)	0.917
Hand-sewn, end-to-end	3 (5.0)	9 (11.0)	0.206
Hand-sewn, side-to-side	5 (8.3)	53 (64.6)	< 0.001
Intra- or extracorporeal anastomosis, *n* (%)			
Intracorporeal anastomosis	31 (51.7)	20 (24.4)	< 0.001
Extracorporeal anastomosis	29 (48.3)	62 (75.6)	
Intraoperative blood loss, *n* (%)			
< 100 ml	118 (81.9)	212 (84.8)	0.459
100–500 ml	18 (12.5)	32 (12.8)	0.931
> 500 ml	8 (5.6)	6 (2.4)	0.103
Duration of surgery in minutes, median (IQR)	125.0 (101.8–180.0)	159.5 (121.2–198.8)	< 0.001
Intraoperative fluid treatment, *n* (%)			
< 500 ml	23 (16.0)	62 (24.8)	0.040
500–1000 ml	62 (43.1)	128 (51.2)	0.119
> 1000 ml	59 (41.0)	60 (24.0)	< 0.001
Intraoperative dexamethasone, *n* (%)	104 (72.2)	117 (46.8)	< 0.001
Perioperative regional analgesia, *n* (%)			
Epidural	89 (61.8)	45 (18.0)	< 0.001
TAP block	10 (6.9)	12 (4.8)	0.372
TQL block	0	2 (0.8)	–

*IQR* interquartile range, *TAP* transversus abdominis plane, *TQL* transmuscular quadratus lumborum

**Table 3:** Postoperative characteristics of patients included in the IBD surgery database

Postoperative characteristics	Non-ERAS (*n* = 144)	ERAS (*n* = 250)	*p* value
Time to first bowel movement in days, median (IQR)	2.0 (1.0–3.0)	2.0 (1.0–2.0)	0.166
Length of stay in days, median (IQR)	6.0 (4.0–8.0)	4.0 (3.0–7.0)	< 0.001
Postoperative complication CD, *n* (%)			
1	3 (2.1)	3 (1.2)	0.490
2	13 (9.0)	34 (13.6)	0.177
3A	3 (2.1)	7 (2.8)	0.663
3B	19 (13.2)	23 (9.2)	0.216
4A	4 (2.8)	5 (2.0)	0.619
4B	0	3 (1.2)	–
5	1 (0.7)	1 (0.4)	0.692
Reoperation, *n* (%)	22 (15.3)	27 (10.8)	0.195
Type of surgical complication requiring reoperation, *n* (%)			
Hemorrhage	1 (0.7)	4 (1.6)	0.439
Surgical site infection	1 (0.7)	0	–
Fascial dehiscence	5 (3.5)	2 (0.8)	0.053
Stoma complication	2 (1.4)	3 (1.2)	0.872
Anastomotic leakage	8 (5.6)	5 (2.0)	0.057
Ileus	2 (1.4)	5 (2.0)	0.658
Other	3 (2.1)	8 (3.2)	0.517
Medical complication, *n* (%)			
Thromboembolic complication	3 (2.1)	5 (2.0)	0.955
High-output ostomy	16 (20.0)	40 (26.0)	0.181
Anastomotic leakage, *n* (%)	6 (10)	8 (9.8)	0.962
Readmission, *n* (%)	23 (16.0)	56 (22.4)	0.125

*IQR* interquartile range, *CD* Clavien-Dindo

### ERAS protocol

From March 2020 onward, patients that underwent surgery for IBD were admitted to a dedicated colorectal surgery unit at our hospital and followed the same pre-, intra-, and postoperative ERAS protocol as those undergoing surgery for colorectal cancer.

IBD-specific amendments included preoperative outpatient counseling with a colorectal surgeon experienced in the surgical treatment of IBD, preoperative intravenous iron for those with iron deficiency anemia, and risk stratification for anastomotic leak in patients with Crohn’s disease to assess whether a patient would benefit most from an anastomosis or a temporary ostomy on the basis of their individual risk factors. For a more detailed description of all items included in our IBD-specific colorectal ERAS protocol, see Table 4.

**Table 4:** Description of items in IBD Enhanced Recovery After Surgery protocols at our department

	Item	Non-ERAS	ERAS
Preoperative	Patient seen by colorectal surgeon experienced in the surgical treatment of IBD for informed consent for surgery	√	√
	Risk stratification for anastomotic leak in patients with CD for preoperative planning of anastomosis vs. ostomy: High risk—systemic steroids, intra-abdominal abscess; Low risk—smoking, emergency surgery, weight loss > 10%, hypoalbuminemia, prior resection. The presence of 1 or more high risk factors will indicate a preference for creating an ostomy over an anastomosis, particularly in case of multiple additional low risk factors. Will always be discussed between the treating colorectal surgeon and the patient. Contraindication for anastomosis: active colonic CD, perianal disease		√
	Nutritional screening and if necessary, protein shakes prescribed. Anemia screening introduced in 2023, with IV iron for patient with iron deficiency anemia prior to surgery		√
	Patient education—weekly information session for patients by a nurse and physiotherapist with information on what to expect after surgery, physiotherapy instructions (1.5 h)		√
	Counseling session with ostomy nurse if there is a chance that an ostomy will be created (1.5 h)		√
	Patient seen by anesthesiologist	√	√
	Patients allowed to eat solids until 6 h and encouraged to drink clear liquids preferably containing carbohydrates until 2 h before surgery	√	√
	Mechanical bowel preparation only for left-sided and rectum resections	√	√
	No standard preanesthetic medications	√	√
Intraoperative	PONV prophylaxis with dexamethasone and/or ondansetron	√	√
	Standard anesthetic protocol using short-acting general anesthetics		√
	Intraoperative intravenous fluid therapy with the goal to maintain euvolemia		√
	Minimal invasive surgery when possible	√	√
	IV antibiotics according to department guidelines	√	√
	Active intraoperative warming to achieve normothermia		√
	No placement of abdominal or pelvic drains unless for specific reasons		√
	Routine use of epidural analgesia in all patients with IBD unless contraindicated	√	
	Planned epidural anesthesia on special indication (chronic pain or planned open surgery)		√
	Transverse incision for specimen removal in UC, midline incision in CD		√
	Local anesthetic administered at incision sites	√	√
Postoperative	No routine use of nasogastric tube unless for postoperative ileus	√	√
	Restrictive intravenous fluid treatment. Treatment only for significant negative fluid balance (e.g.,, high output, paralytic ileus with fluid loss through nasogastric tube) after discussion with the treating colorectal surgeon		√
	Removal of urinary catheter on admission to PACU unless otherwise specified		√
	Early mobilization upon arrival at postoperative ward, time out of bed > 8 h per day		√
	Thromboprophylaxis according to department guidelines with compression stockings & heparin	√	√
	Use of postoperative multimodal analgesia	√	√
	Reintroduction of solid food on the evening of POD 0. If oral intake is insufficient at POD 3, nutritional screening is performed at POD 4 for consideration of parenteral nutrition		√
	Reintroduction of solid food on the evening of POD 0	√	
	Use of chewing gum for reduction of paralytic ileus		√
	Use of positive expiratory pressure devices for respiratory therapy		√
	Admission to colorectal surgery ward with nurses trained in ERAS and caring for patients with IBD		√
	Patients admitted to the general surgery postoperative ward	√	
	Rectal stump washout from POD 2 until discharge using catheter on special indication only		√
	72 h antibiotic prophylaxis for all patients after subtotal colectomy		√
	Daily measurement of stoma output, with focus on high-output syndrome		√
After discharge	Phone call from nurse 2–3 days after discharge		√
	Regular outpatient clinic follow-up with stoma nurse to ensure proper stoma function and care		√
	Outpatient follow-up by gastroenterologist for assessment of postoperative medication for IBD	√	√
	Quarterly audits of surgical trajectories with specifically set quality indicators		√

*ERAS* Enhanced Recovery After Surgery, *IBD*   inflammatory bowel disease, *MDT* multidisciplinary team, *CD* Crohn’s disease, *UC* ulcerative colitis, *POD* postoperative day, *PACU*  post anesthesia care unit

While not a classical part of ERAS, optimal patient treatment was furthermore discussed at a weekly IBD multidisciplinary team (MDT) meeting and patients were followed up by a gastroenterologist after discharge to assess for the postoperative necessity for IBD medications.

### Outcomes

Our primary outcome was the occurrence of a serious complication, defined as a complication of Clavien-Dindo grade 3 or higher [12]. If a patient suffered from more than one complication, the highest grade was registered.

We furthermore examined postoperative length of stay (LOS) in days and whether a patient was readmitted as secondary outcomes.

We performed subgroup analyses for patients with Crohn’s disease and ulcerative colitis separately, as well as for repeat surgeries for IBD and elective surgeries, which we considered to be the group with the highest adherence to our ERAS protocol.

A sensitivity analysis was furthermore performed to assess the impact of minimal invasive surgery before and after the implementation ERAS and whether it was associated with changes in postoperative LOS.

### Statistical analysis

Summary statistics were compiled, with median and interquartile range used for continuous data and frequency and percentages used for categorical data. We compared continuous data using Mann–Whitney* U* test and categorical data using χ^2^ test and considered a *p* value < 0.05 to be statistically significant. We calculated odds ratios (OR) using logistic regression with the R packages ‘stats’ with a confidence interval (CI) set at 95%, and an adjusted odds ratios (aOR) where we adjusted for sex and age.

All statistical analyses were performed using R version 4.3.1.

This study was approved by the Danish Data Protection Agency (REG-093-2020). Retrospective review of medical records was approved by the Danish Patient Safety Authority (STPS: 31-1521-451) prior to July 2020 and individual written consent by patients from July 2020 onward. Study data were entered into a database using REDCap electronic data capture tools hosted at Zealand University Hospital [13]. This manuscript was prepared according to the STROBE guidelines [14].

## Results

Between December 1, 2017 and August 30, 2023, a total of 468 patients were operated on for inflammatory bowel disease at Zealand University Hospital. We excluded 70 patients that underwent emergency surgery and 4 patients who did not give consent for use of their data for research purposes, resulting in 394 patients that were included in this study. Of these, 144 patients were operated on before the implementation of ERAS and 250 patients after the implementation of ERAS (Tables 1, 2, 3).

The occurrence of serious postoperative complications remained similar under ERAS (15.6%) vs. non-ERAS (18.8%, *p* = 0.420) with an OR of 0.80 (95% CI 0.47–1.39) and an aOR of 0.73 (95% CI 0.42–1.28).

LOS decreased from a median of 6 days to a median of 4 days (*p* < 0.001) in the ERAS cohort, with no higher rate of readmissions under ERAS (22.4%) vs. non-ERAS (16.0%, *p* = 0.125) (Tables 1, 2, 3).

### ERAS in Crohn’s disease

A total of 150 patients with Crohn’s disease were operated on under ERAS protocols compared to 102 prior to the introduction of ERAS. The occurrence of serious postoperative complications remained similar under ERAS (14.7%) vs. non-ERAS (14.7%, *p* = 0.993), with an OR of 1.00 (95% CI 0.49–2.06) and an aOR of 0.96 (95% CI 0.47–1.99).

LOS decreased from a median of 5 days to a median of 4 days (*p* = 0.011) in the ERAS cohort, but with a higher rate of readmissions under ERAS (22.7%) vs. non-ERAS (10.8%, *p* = 0.016).

### ERAS in ulcerative colitis

After the introduction of ERAS, 98 patients were operated on for ulcerative colitis compared to 40 patients prior to the introduction of ERAS. The occurrence of serious postoperative complications decreased non-significantly under ERAS (16.3%) vs. non-ERAS (27.5%, *p* = 0.133), with an OR of 0.51 (95% CI 0.21–1.26) and an aOR of 0.44 (95% CI 0.18–1.12).

LOS decreased from a median of 6 days to a median of 4 days (*p* = 0.007) in the ERAS cohort, with no higher rate of readmissions under ERAS (21.4%) vs. non-ERAS (30%, *p* = 0.284).

### ERAS in elective surgery

When only elective surgery was analyzed, a total of 172 patients underwent surgery for IBD after the introduction of ERAS compared to 99 patients prior. The occurrence of serious postoperative complications remained similar under ERAS (12.8%) vs. non-ERAS (14.1%, *p* = 0.752), with an OR of 0.89 (95% CI 0.44–1.87) and an aOR of 0.79 (95% CI 0.38–1.68).

LOS decreased from a median of 5 days to a median of 4 days (*p* = 0.010) in the ERAS cohort, with a higher rate of readmissions under ERAS (22.7%) vs. non-ERAS (12.1%, *p* = 0.032).

### ERAS in repeat surgeries for IBD

When only patients who underwent repeat surgeries for IBD were analyzed, a total of 85 patients underwent repeat surgery for IBD after the introduction of ERAS compared to 61 patients prior. The occurrence of serious postoperative complications remained similar under ERAS (18.8%) vs. non-ERAS (16.4%, *p* = 0.705), with an OR of 1.18 (95% CI 0.50–2.90) and an aOR of 1.02 (95% CI 0.42–2.56). LOS decreased from a median of 5 days to a median of 3 days (*p* = 0.021) in the ERAS cohort, with a similar rate of readmissions under ERAS (23.5%) vs. non-ERAS (16.4%, *p* = 0.293).

### Sensitivity analysis of minimal invasive surgery

After the implementation of ERAS, significantly more patients underwent surgery using a minimally invasive approach than before (90.4% vs. 83.3%, *p* = 0.039), with even fewer conversions to open surgery (4.4% vs. 17.5%, *p* < 0.001). In order to examine whether it was solely the effect of an increased use of minimally invasive surgery on shortened postoperative LOS, we analyzed a subgroup of patients undergoing laparoscopic surgery before and after the introduction of ERAS. After ERAS was introduced, 226 patients underwent surgery for IBD compared to 120 patients before. The occurrence of serious postoperative complications remained similar under ERAS (15.0%) vs. non-ERAS (20.8%, *p* = 0.173), with an OR of 0.67 (95% CI 0.38–1.20) and an aOR of 0.60 (95% CI 0.28–1.08).

LOS decreased from a median of 6 days to a median of 4 days (*p* < 0.001) in the ERAS cohort. This was still the case when we removed patients in whom conversion to open surgery was necessary (6 days vs. 4 days, *p* = 0.001). There was no higher rate of readmissions under ERAS (21.7%) vs. non-ERAS (15.0%, *p* = 0.134).

## Discussion

We can demonstrate that utilizing an IBD-specific ERAS pathway in patients undergoing surgery for IBD resulted in no differences in postoperative complications of Clavien-Dindo grade 3 or higher. Median LOS decreased from 6 to 4 days, with similar rates of readmissions. A sensitivity analysis showed that it was not solely the effect of an increased use of minimal invasive surgery that was associated with a reduced postoperative LOS.

While other studies have shown similar reductions in LOS after the introduction of ERAS in surgery for IBD, none have reported an IBD-specific ERAS pathway [15–19]. Preoperative counseling with an IBD surgeon in the outpatient clinic, intravenous iron treatment for patients with iron-deficiency anemia, and preoperative risk stratification for anastomotic leakage were some of the items added to our department’s ERAS protocol utilized for patients undergoing surgery for colorectal cancer.

Despite these IBD-specific additions, there are further items that might be useful such as discussing timely withdrawal of specific IBD medications prior to surgery. While the literature on biological therapy and postoperative complications is conflicting and most likely use of tumor necrosis factor (TNF) alpha inhibitors is safe, the use of steroids is associated with a higher risk for infectious and non-infectious complications [20–23]. With 32% of patients in our ERAS cohort using systemic corticosteroids in the month up to surgery, this suggests that steroid treatment reduction or withdrawal should be discussed with the gastroenterologist at the preoperative MDT meeting.

Additionally, personalized multimodal prehabilitation strategies could improve postoperative outcomes further. While exercise to increase functional capacity before surgery might not be as impactful as in patients operated on for colorectal cancer because of inherent differences in age, future IBD-ERAS protocols will almost certainly incorporate nutritional prehabilitation, smoking cessation programs, and consultations with psychologists, physiotherapists, and dietitians [24–27].

It is important to note that we have removed the systematic use of epidural analgesia from our ERAS protocols as we did not see better pain relief but rather prolonged recovery time in our patients. While epidural analgesia is classically a part of ERAS, owing to its ability to decrease the surgical stress response, evidence of its advantages is lacking in minimally invasive surgery [28]. In select patients that either have a high risk of requiring conversion to open surgery or are known to suffer from chronic pain, placing a preoperative epidural is discussed between the patient and the treating anesthesiologist.

We found a slight increase in readmissions that was significant on subgroup analysis in patients with Crohn’s disease and patients undergoing elective IBD surgery. This might stem from two practice changes that occurred around the time that ERAS was implemented in our department for patients with IBD. In our department, a nurse calls patients 2–3 days after discharge as a means of follow-up. Also in the Danish healthcare system, a rule was introduced that patients within the first 72 h of being discharged can contact the discharging department directly in case of problems. Prior to that, these patients had to be seen by their general practitioner before being sent for readmission to the hospital. We believe that both changes have lowered the bar for getting readmitted, while the rate of complications has remained similar. We only counted a true readmission when a patient stayed overnight in order to mitigate this difference.

Limitations of this study include its observational single-center design and resulting limited sample size, a non-contemporaneous control bias, and that we did not have data on compliance with ERAS protocols for every item or patient. We performed a subgroup analysis on patients undergoing only elective surgery for IBD, which we considered the patient group with the highest compliance with ERAS protocols, which showed similar results. Moreover, anemia screening with intravenous iron infusion for those with iron deficiency anemia was introduced within the previous year, which could have impacted results. While the use of chewing gum for the prevention of postoperative paralytic ileus is controversial, we have decided to keep this item in our ERAS protocol as we believe that it is a low cost and low risk intervention [29, 30]. We furthermore did not assess patients that underwent emergency surgery for IBD, as these patients followed a different emergency surgery bundle care pathway in our department. However, we decided to include patients that underwent subacute surgery for IBD, as we believe that these patients still benefit from ERAS pathways despite not following all preoperative ERAS items stringently. It is especially this patient group that has a high risk for complications and long postoperative LOS. Further, we believe that while LOS is an objective indicator of patient recovery, using patient-reported outcome measures (PROM) such as the Quality of Recovery Index-15 (QOR-15) would be superior in this context, both in-hospital and post-discharge [31].

## Conclusion

We demonstrate that an IBD-specific ERAS pathway can successfully be applied in surgery for patients with inflammatory bowel disease. The use of ERAS was associated with a reduced postoperative LOS, but no impact on the occurrence of serious complications or the rate of readmissions.

## Data Availability

The data underlying this article cannot be shared publicly due to privacy of individuals that participated in the study and the European Union’s General Data Protection Regulations*. *Anonymized data will be shared on reasonable request to the corresponding author.
